# Piezoelectric Peptide Nanotube Substrate Sensors Activated through Sound Wave Energy

**DOI:** 10.1021/acsmaterialslett.3c01613

**Published:** 2024-04-08

**Authors:** Sawsan Almohammed, Allan Finlay, Dominik Duleba, Shane Cosgrave, Robert Johnson, Brian J. Rodriguez, James H. Rice

**Affiliations:** † School of Physics, University College Dublin, Belfield, Dublin 4 D04 V1W8, Ireland; ‡ Conway Institute of Biomolecular and Biomedical Research, University College Dublin, Belfield, Dublin 4 D04 V1W8, Ireland; § School of Chemistry, University College Dublin, Belfield, Dublin 4 D04 V1W8, Ireland

## Abstract

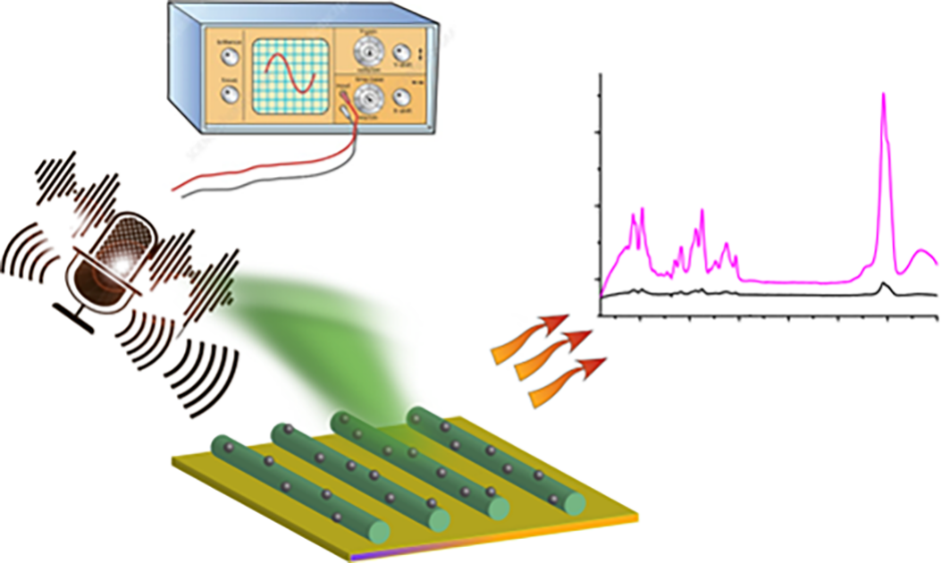

The use of sustainable and safe materials is increasingly in demand for the creation of photonic-based technology. Piezoelectric peptide nanotubes make up a class of safe and sustainable materials. We show that these materials can generate piezoelectric charge through the deformation of oriented molecular dipoles when the tube length is flexed through the application of sound energy. Through the combination of peptide nanotubes with plasmon active nanomaterials, harvesting of low-frequency acoustic sound waves was achieved. This effect was applied to boost surface-enhanced Raman scattering signal detection of analytes, including glucose. This work demonstrates the potential of utilizing sound to boost sensing by using piezoelectric materials.

Piezoelectric nanogenerators (PENGs) are a device class that converts mechanical energy into electrical energy using the piezoelectric effect.^1,2^ PENGs have been made from a range of organic and inorganic materials such as barium titanate, polyvinylidene fluoride (PVDF), and γ-glycine.^1,3,4^ When mechanical vibrations or movements are applied to the PENG device, the piezoelectric material can deform, generating an electric charge. This generated charge can then be collected and used as electrical energy. PENG nanogenerators can be potentially applied in a range of areas, such as in structural health monitoring or self-powered sensing.^1,2^ Monitoring the health of bridges or buildings is done by converting structural vibrations into electrical power for sensing equipment or harnessing industrial machinery vibrations and noise for powering (environmental) sensors or other low-power devices.

In this work, we show that a PENG nanogenerator can boost sensing signal sensitivity by harvesting acoustic energy. We show that surface-enhanced Raman scattering (SERS) based sensing can be boosted by over a magnitude using a PENG device when acoustic energy is applied. SERS is an ultrasensitive analytical method widely used in areas such as environmental monitoring and food safety.^5,6^ Applying mechanical stress to a PENG nanogenerator the piezoelectric active material induces aligned dipole moments and charge transfer creating an electric potential.^2^ Such piezoelectric fields can efficiently control electron densities around plasmon active metal nanostructures which can increase SERS signal strengths by enhancing the electromagnetic fields generated by the plasmonic nanostructure.^5^ Additionally, the piezoelectrically generated charge transfer processes can enhance SERS through the chemical enhancement mechanism.^7,8^ Piezoelectric field up-regulated surface-enhanced Raman spectroscopy (E-SERS) is reported to effectively increase Raman signal intensities.^9,10^ Studies have shown that combining PVDF acting as the piezoelectric-modulated layer and silver nanowires which act as the SERS active layer enhances E-SERS signals (>2-fold) under applied stress.^9,11,12^ Applying stress to the piezoelectric modulates the PVDF internal electric field, which can effectively regulate the surface plasmonic property of the silver nanowires. This in turn modulates the distribution of the electric field around the silver nanowires, causing an enhancement in SERS signal strength.

The use of sustainable and safe materials is increasingly in demand for the creation of photonic-based technology. Policy initiatives such as the new Chemicals Strategy for Sustainability in the European Union and the support for green innovation and technology by the Organisation for Economic Co-operation and Development aim to encourage the use of such materials in industry.^13^ Bioinspired materials are attractive in this regard, as they can be both sustainable and safe materials. Peptide nanotubes are a class of bioinspired materials that are piezoelectric with a d_33_ value (18 pm/V) which is comparable to well-known piezoelectric materials such as zinc oxide (11.7 pm/V) and PVDF nanofibers (ε_33_ = 30 pm/V).^14^ It has been shown that piezoelectric materials quasi-1D peptide nanotubes combined with plasmonic metal nanomaterials support E-SERS.^6^ E-SERS was induced by applying mechanical stress to the peptide nanotube and silver nanoparticle sample. Strengthening SERS signal intensities by over an order of magnitude for a range of molecules including albumin, lysozyme, glucose, and adenine.^6^ Sound waves represent an energy source that can be potentially harvested by piezoelectric materials.^15^ The most ubiquitous environmental noise is low frequency (<500 Hz).^15^ Here, we show that combining peptide nanotubes with plasmon active nanomaterials can harvest low-frequency acoustic sound waves (<500 Hz) to support E-SERS. Generating piezoelectric charge through sound wave induced deformation of the nanotubes longitudinal oriented molecular dipoles. This effect was applied to boost SERS signal detection of analytes including glucose. This work demonstrates the potential of utilizing sound to boost SERS-based sensing using a PENG device.

A thin film peptide nanotube/plasmon active silver nanoparticle composite was prepared following a reported procedure and as outlined in the experimental section.^16,17^ The nanocomposite formed from a single layer of peptide nanotubes on top of the nanotubes is present a layer of plasmon active nanoparticles. The thin film peptide nanotube/plasmon active silver nanoparticle composite was formed between gold electrodes on a silicon oxide/silicon substrate (shown schematically in Figure 1a). The nanotubes were formed aligned on the silicon oxide region using the wettability difference between silicon and silicon dioxide.^16^ Silver nanoparticles are then deposited onto the nanotubes to form a piezoelectric-plasmonic hybrid composite. The working operation of the peptide nanotube/silver nanoparticle composite substrate is schematically outlined in Figure 1b. This model is based on experimental studies and COMSOL simulations, as outlined below. We propose that sound, including ambient sound frequencies, can be harvested by the PENG device. The acoustic energy stimulates the piezoelectric potential in the peptide nanotube. This results in a charge build up on the surface of the nanotube that can cause charge to transfer to the silver nanoparticle, boosting the SERS signal from analyte molecules such as glucose present on the plasmon active metal nanoparticles.

**Figure 1:**
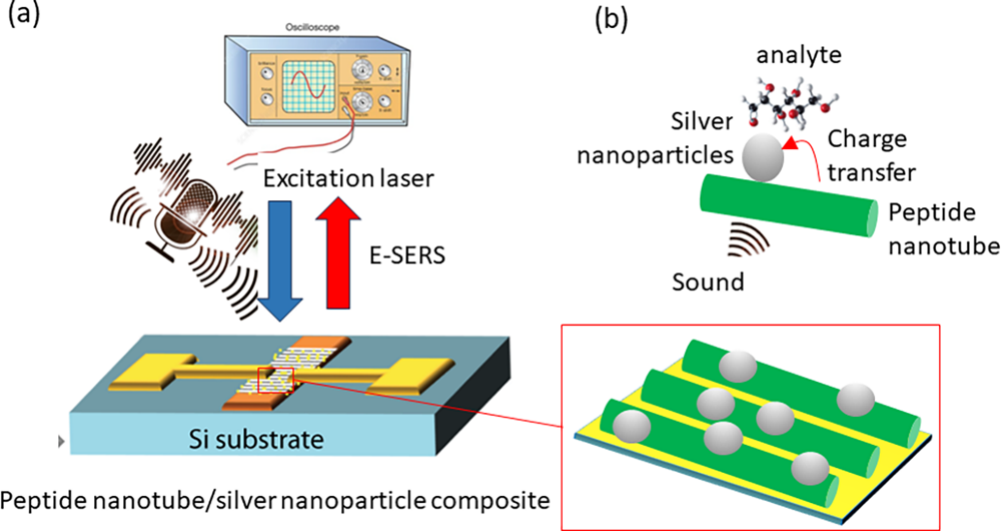
Structural design and operating principle of the peptide nanotube/silver nanoparticle substrate. (a) Schematic of the substrate showing gold electrodes (yellow) formed on a silicon substrate (blue) with a region between the electrodes (orange) being silicon oxide. The aligned peptide nanotubes and silver nanoparticle composite are located in the orange region. (b) Schematic illustration of the working operation of the peptide nanotube/silver nanoparticle composite substrate. Sound including ambient sound frequencies stimulates a piezoelectric potential in the peptide nanotube, causing charge to transfer to the silver nanoparticle supporting E-SERS from analyte molecules present on the plasmon active metal nanoparticle.

The substrate was prepared following a reported procedure (outlined in Supporting Information).^16,17^ Scanning electron microscopy (SEM) images (Figure 2a–c) show the aligned peptide nanotube–silver nanoparticle composite in the gap between the gold electrodes. The silver nanoparticle aggregates on the peptide nanotubes interact with the nanotube via functional groups on the surface of the tube.^16^ This causes them to pack together to form a quasimonolayer on the surface of the nanotubes (Figure 2c). SEM imaging shows that the diameter of the peptide nanotubes was 3.5 ± 1 μm from *n* = 30 nanotubes. The average distance between the silver nanoparticles was estimated to be 80 ± 30 nm (*n* = 60), in agreement with previous reports.^16,17^ Optical micrographs (as shown in Supporting Information Figure S1) were used to confirm alignment through visual inspection. Quantimeter of the alignment of the nanotube-nanoparticle composite through using the radial summation of the fast Fourier transform (FFT) of the optical image (Figure 2d and Supporting Information S1) was undertaken. The degree of alignment was assessed as the average peak full width at half-maximum (fwhm) of the radial summation of the FFT of the optical images (from five images for each sample type), determined by Gaussian fit, as employed previously.^16^ The fwhm decreased with increased alignment. A value of fwhm (full width at half-maximum) of 20 ± 1° was determined from the plot. This is compared with literature values that show that unaligned peptide nanotubes possess a fwhm of ca. 70 ± 1°. Lateral piezoresponse force microscopy (LPFM) phase images of the peptide nanotubes (as shown in Supporting Information Figure S2) show that the peptide nanotubes possess a high degree of uniformity of polarization.

**Figure 2:**
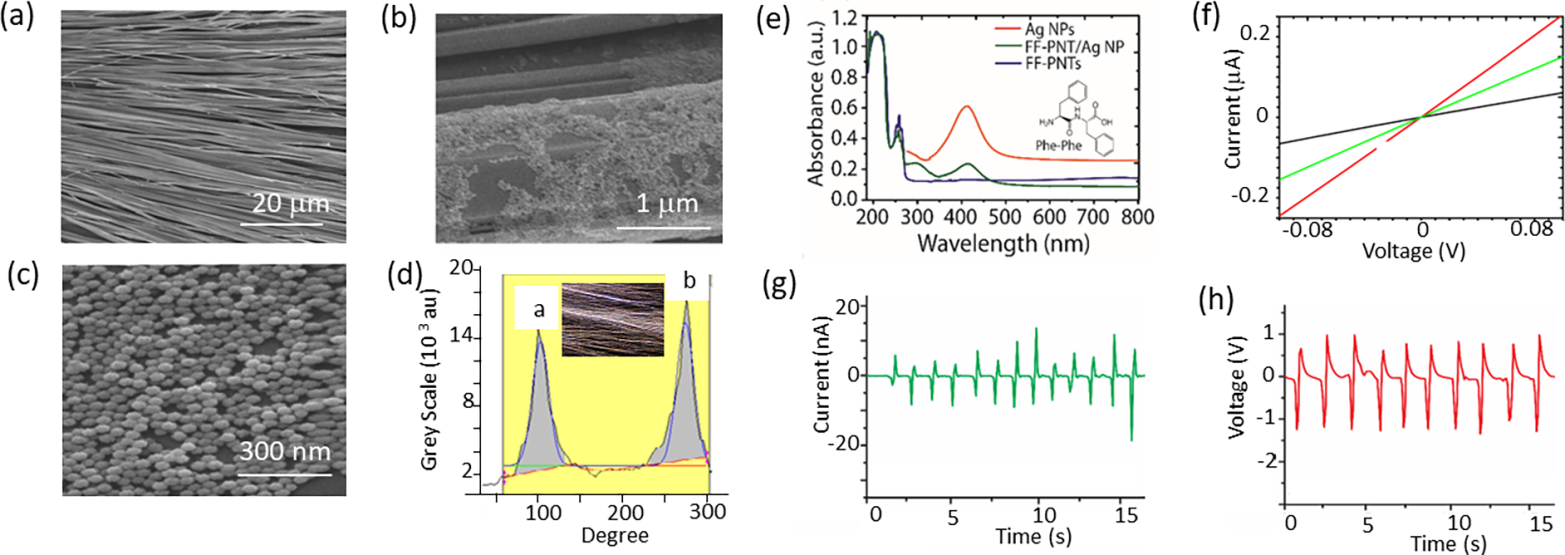
(a–c) Scanning electron imaging (SEM) images of the aligned peptide nanotube–silver nanoparticle composite recorded at different image resolutions. (a) Shows the aligned peptide nanotubes. (b) Zooms into a single nanotube to show the presence of silver nanoparticles on the surface of the nanotube. (c) High image resolution image of the surface of a single peptide nanotube showing the presence of nanoparticle clusters. These nanoparticles are present in a close packing-like arrangement on the surface of the nanotubes. (d) Radial summation of the FFT intensity of an optical image of aligned nanotubes (shown as an insert image) versus angle showing Gaussian fits (blue line). The fit was used to determine the fwhm, i.e., the degree of alignment. (e) Optical absorption spectra of silver nanoparticles (red line Ag NPs), peptide nanotubes (purple line FF-PNTs) only and peptide nanotubes combined with silver nanoparticles (green line FF-PNT/Ag NPs). (f) IV plot for silver nanoparticles (black line Ag NPs), peptide nanotubes (red line FF-PNTs) only and peptide nanotubes combined with silver nanoparticles (green line FF-PNT/Ag NPs). (g,h) Current and open-circuit voltage output over time from the peptide nanotube–silver nanoparticle template obtained by bending the substrate.

The optical absorption spectrum of the silver nanoparticles shows only a band at 420 nm corresponding to a localized surface plasmon resonance (LSPR) (Figure 2e). This band possesses a full width at half-maximum (fwhm) of 45 nm. While the absorption spectrum for peptide nanotubes shows absorption bands only at 220 and 260 nm (Figure 2e). Combining the peptide nanotubes with silver nanoparticles causes the silver nanoparticles’ LSPR feature to shift by 15 to 435 nm. Along with this red-shift, the LSPR bands fwhm narrowed by 15 to 30 nm (Figure 1b). This change in the position and fwhm of the LSPR band has been reported to arise from the peptide amino acid carboxyl groups of the peptide nanotubes strongly binding to the silver nanoparticles.^3,16^ Current–voltage (IV) measurements of the nanotube-nanoparticle substrate show Ohmic behavior (Figure 2f) with a resistance of 660 Ω measured. This value has been lower than for silver nanoparticles (1580 Ω) or peptide nanotubes (930 Ω) alone. This is in line with previous studies that report a lowering of the resistance of peptide nanotubes upon incorporation of metal nanoparticles.^18^ Open-circuit voltages and short-circuit currents were obtained by bending peptide nanotubes aligned on a flexible polymer substrate. Current and voltage outputs showed negative followed by positive current and voltage outputs (Figure 2g, h) in agreement with previous studies.^19^ The average voltages were −0.8 ± 0.1 and 1.3 ± 0.2 V (current output was −7 ± 0.7 nA and 5 ± 0.7 nA) for the negative and positive current peaks, respectively.

We then undertook studies to examine the potential of the peptide nanotube–silver nanoparticle substrate to enhance Raman scattering signal intensities through harvesting sound energy. Recording the E-SERS spectra were recorded when applying sound with an incident sound pressure of 100 dB and varying the sound frequencies, fixing the microphone-sample distance to 1 cm. The application of sound intensities of 100 dB (the maximum sound intensity of the device) was required to generate a change in Raman signal intensity (Figure 3a inset). Applying sound intensities lower than this resulted in no SERS signal boosting through the application of sound. A digital function generator was used to accurately control the sound frequency from 10 to 2000 Hz with a step of >25 Hz. The E-SERS spectra of para-aminothiophenol (PATP) on the substrate recorded at 10 Hz (Figure 3a) shows A_1_ modes at 1080 and 1594 cm^–1^ with further peaks observed at 1145, 1390, and 1437 cm^–1^. These peaks (at 1145, 1390, and 1437) can be attributed to b_2_ symmetry arising from the formation of PATP dimers, e.g., 4,4′-dimercaptoazobenzene (DMAB).^3,7^ Fixing the sound pressure at 100 dB and increasing the sound frequency (10 to 2000 Hz) were seen to change the observed E-SERS spectra (Figure 3a, b). Applying low-frequency sound of 10 to 250 Hz increased the E-SERS signal intensity ca. 2-fold (Figure 3a, b). Increasing the sound frequency further (300 to 2000 Hz) resulted in a ca. 1.5-fold increase in E-SERS signal intensity.

**Figure 3:**
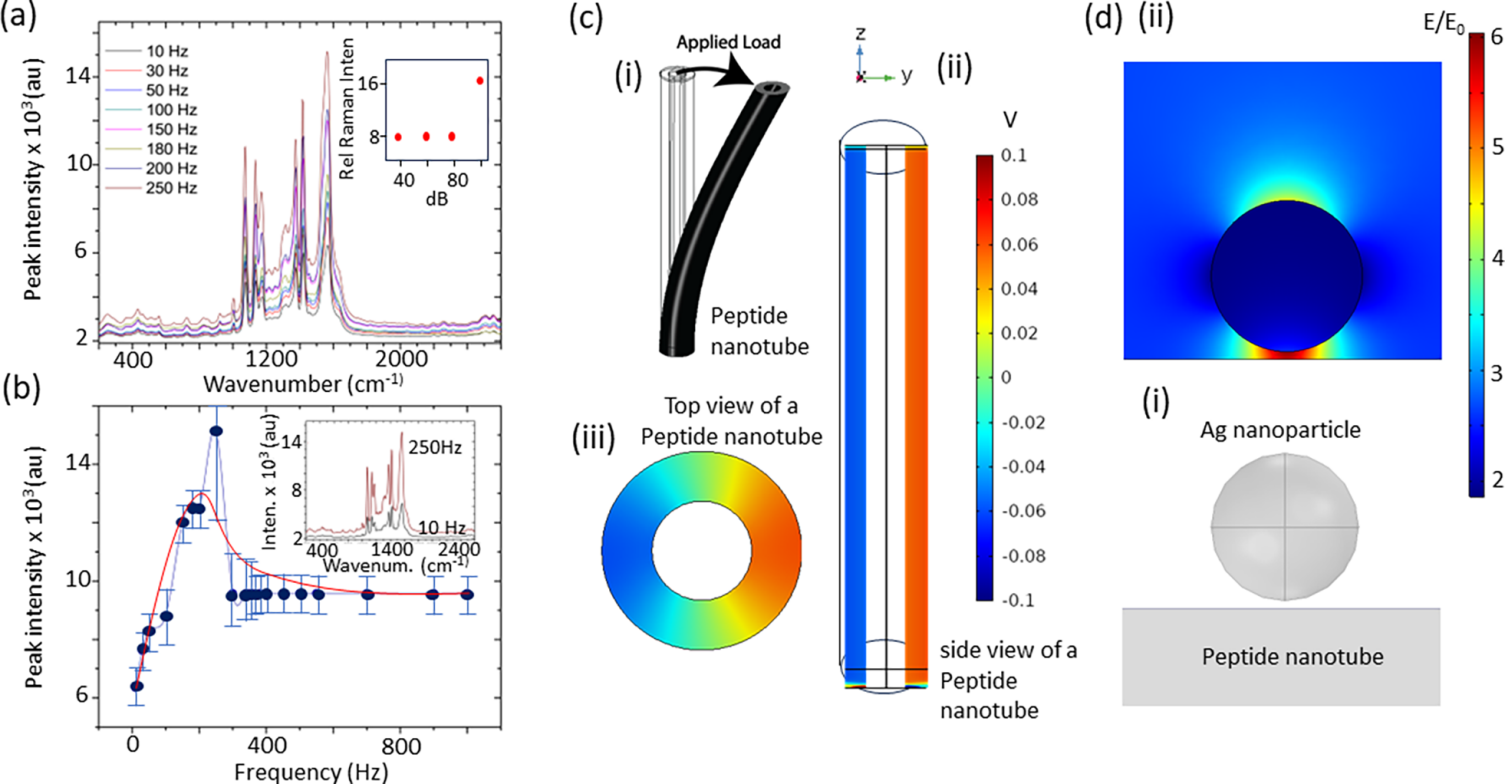
(a) SERS spectra of PATP on peptide nanotubes combined with silver nanoparticles recorded as a function of sound frequency 10 to 250 Hz). Inset showing a plot of sound intensity (dB) versus SERS signal intensity. (b) Plot of SERS intensity as a function of sound frequency. (c) (i) Schematic drawing of a peptide nanotube with sound waves striking the side of the nanotube that applies a load and results in the bending of the nanotube. The nanotube model diameter was set to 350 nm with a hollow core of 175 nm, and the length was set to 30 μm. (ii) Side-view of the stimulated peptide nanotube’s piezoelectric potential (*z*-axis scale is set to 0.1 for visualization). (iii) Top-view of the nanotube showing the stimulated piezoelectric potential. (d) The electric field distribution of a silver nanoparticle placed on a peptide nanotube as shown schematically in (i). (ii) Simulated electric field on a silver nanoparticle generated by the piezoelectric potential of the peptide nanotube.

Studies have shown that a range of organic materials can generate electrical power through harvesting sound energy.^20,21^ It has been shown that applying sound to PVDF nanofibers with an intensity of ca. 100 dB and frequencies of ca. 200 Hz on PVDF fibers can cause compression of the fiber resulting in piezoelectric charge formation.^22^ The Young’s Modulus for the peptide nanotubes (19 GPa) are comparable to PVDF (4 GPa).^22,23^ This indicates that deformation of the peptides can be similarly achieved for PVDF as stimulated through acoustic wave energy. To give a deeper insight into this phenomenon, COMSOL Multiphysics modeling was applied. First, a single nanotube was modeled (as shown schematically in Figure 3c-i) using parameters outlined in Supporting Information. Then simulations of compression of the nanotube through the application of a 10 nm transverse load were performed. The results from these simulations showed that voltages of ±0.1 V were generated with the potential distributed equally along the tube (Figure 3c-ii). With a positive potential formed in the direction of the applied force (Figure 3c-ii,iii). These surface charges arise from the piezoelectric potential of the peptide nanotube activated through the application of a mechanical force. How these surface charges formed on the peptide nanotube influenced silver nanoparticles’ electric field was then simulated (Figure 3d-i). Simulations show that the electric field in the gap area between the nanoparticle and peptide nanotube and the area atop the nanoparticle is enhanced strongly (Figure 3d-i)) and occurs through charge transfer between the piezoelectric nanotube and the plasmonic nanostructure. The simulations show that when silver nanoparticles close to the peptide nanotube are charged under the electrostatic induction of a negative potential, the electric field around the nanoparticle is enhanced. This in turn strengthens the electromagnetic enhancement mechanism that yields SERS. In addition to the increase in total E-SERs signal intensity, changes in the Raman spectra occurred. The SERS spectra recorded at 250 Hz showed an increase in the relative Raman peak intensity at 1330 cm^–1^ when compared to the E-SERS spectra recorded when 10 Hz was applied. The Raman peak at 1330 cm^–1^ is assigned to the NO_2_-stretching from para-nitrothiophenol (PNTP).^3,7^ Such a redox catalysis reaction has been reported for silver nanostructures on semiconductors.^7,24,25^ Where the semiconductor generates charge through photoexcitation or piezoelectric potentials that transfer to the silver nanoparticle. Resulting in an increase in hot electron populations in the plasmonic nanomaterial. These hot electrons then transfer to oxygen forming singlet oxygen species that then oxidase the target molecule catalyzing PATP to PNTP transformation.^7,24,25^ This effect occurs stimulatingly with the increase in the local electromagnetic field around the silver nanoparticle, enhancing SERS signal intensities from charge transfer from the piezoelectric peptide semiconductor.

The effect of sound frequency on the peptide nanotube with silver nanoparticles present was seen to be significant (Figure 3b) where the highest SERS signal intensity was seen for frequencies at 250 Hz (with sound intensity at 100 dB). Studies of PVDF have reported that piezoelectric charge generation is optimized under acoustic waves with frequencies less than 400 Hz with a sound intensity of 100 dB. This was interpreted to arise from the polymer nanofibers’ flexibility enabling them to vibrate with applied acoustic wave energy.^15^ Potentially the low-frequency sound waves (ca. 200 Hz) match the tube’s average diameter when propagating in the form of plane wave in the tube.^15^ Where open circuit output voltage was highest when the sound frequency was below 400 Hz. Moreover, a response with frequency grew from 0 to ca. 200 Hz and then declined until ca. 800 Hz when it became unchanging with frequency. This is a similar profile response as seen with how peptide film E-SERS intensity responded for PATP with frequency (Figure 3b). Noting the impact of the substrate dimensions and mechanical properties, such as elastic modulus, of silicon, peptide nanotubes, and gold electrodes will also influence the response of the peptides to sound.

We applied the peptide nanotube with a silver nanoparticle substrate to detect glucose, boosting the SERS signal intensity via the application of sound (Figure 4). Detection of glucose is the essential method to monitor the state of diabetes. Conventionally, to measure the glucose in patients’ blood is drawn from patients which can cause discomfort or medical complications in patients such as those with blood disease or blood clotting deficits. There is a need to develop noninvasive or minimally invasive methods for frequent glucose monitoring. SERS detection of glucose has the potential to detect glucose in saliva or sweat but is limited by the low Raman scattering cross-section of the glucose molecule as well as the poor affinity of glucose molecules to be adsorbed on metal surfaces.^26^ We applied the peptide nanotube with a silver nanoparticle substrate to detect glucose, using an applied acoustic sound wave to boost the SERS detection signal (Figure 4). The spectra show the Raman peaks characteristic of glucose.^27^ The major Raman peaks that appeared at 911 cm^–1^, 1060 cm^–1^, and 1125 cm^–1^ are considered to be the Raman fingerprints of glucose. Following the application of an acoustic energy source, the SERS signal increases (Figure 4). The strongest SERS signals are seen around 200 Hz, as was seen for the SERS spectra from probe molecule PATP (Figure 4). The increase in SERS signal arising from the peptides piezoelectric fields efficiently controlling electron densities around plasmon active metal nanostructures potentially increasing SERS signal strengths by enhancing the electromagnetic fields generated by the plasmonic nanostructure.^5^ The work function of peptide nanotubes is 6.2 eV,^28^ based on previously reported Kelvin probe force microscopy (KPFM) data. As the Fermi energy level of the silver nanoparticles is 4.26 eV,^28^ the movement of electrons from the peptide nanotube to the silver nanoparticles is possible, as illustrated in a proposed metal–semiconductor junction band structure for silver and peptide nanotubes (Figure 5). The addition of sound energy at 200 Hz can increase the piezoelectrically generated charge transfer processes by increasing the number of charges moving from the nanotube to the silver, which increases the SERS signal intensities. Increasing the electron densities around plasmon active metal nanostructures increases the SERS signal strengths via better optimizing the electromagnetic fields generated by the plasmonic nanostructure.^6^ The charge can additionally transfer to the probe molecule, which can lead to increased SERS signal intensities via the chemical enhancement factor.^7^

**Figure 4:**
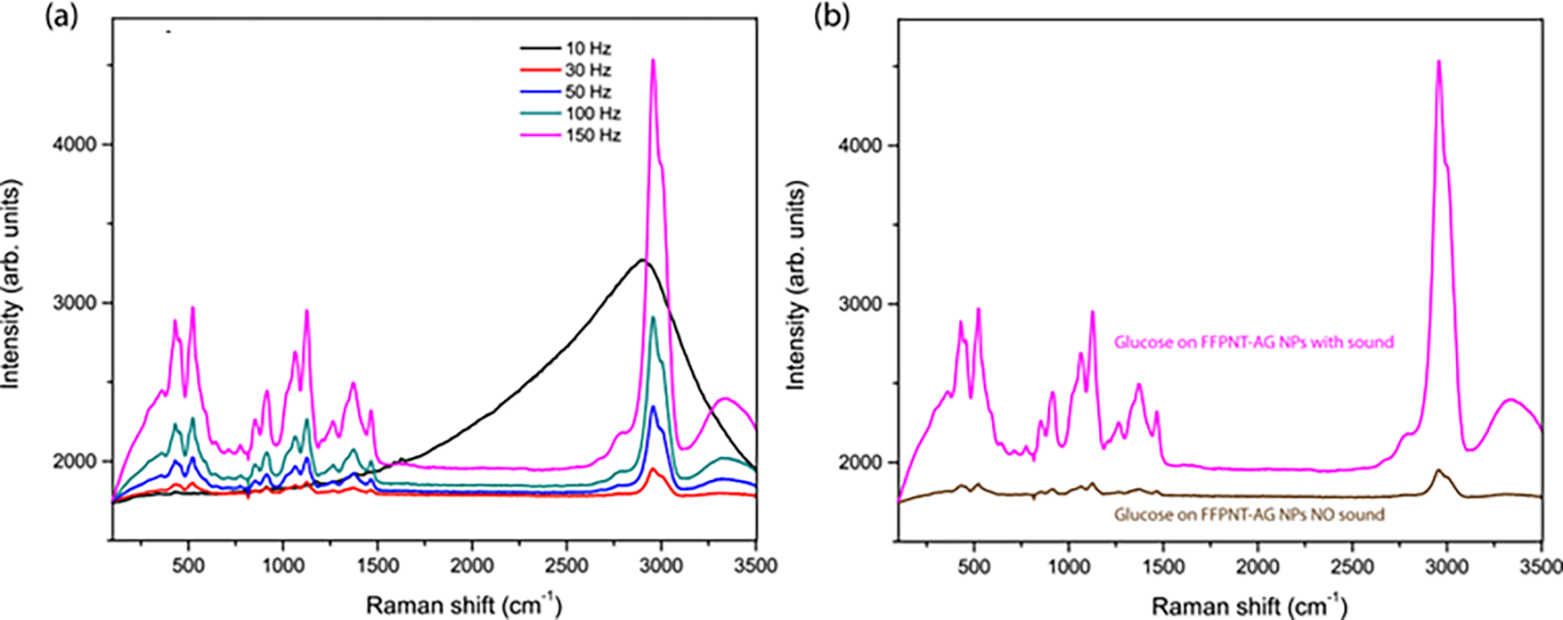
(a) SERS spectra of glucose on peptide nanotubes combined with silver nanoparticles recorded as a function of sound frequency 10 to 250 Hz). Inset, plot of SERS intensity as a function of sound frequency. (b) SERS spectra of glucose on peptides nanotubes combined with silver nanoparticles recorded with no sound frequency applied and with 150 Hz applied.

**Figure 5:**
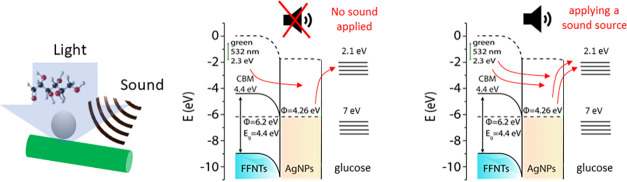
Schematic showing the band diagram of peptide nanotubes (FFNTs) and silver nanoparticle (AgNP) junction/interface. The left-hand band diagram shows before sound energy is applied and the right-hand side shows after sound energy is applied. The addition of sound increases the charge transfer flow as indicated by the red arrows.

In conclusion, we show that combining peptide nanotubes with plasmon active nanomaterials can harvest low-frequency acoustic sound waves to support E-SERS. Finite element simulations show that piezoelectric charge can be generated through induced deformation of the nanotube’s longitudinal oriented molecular dipoles, where such deformation can be induced by sound waves. This effect was applied to boost surface-enhanced Raman scattering signal detection of analytes including glucose. This work demonstrates that peptide nanomaterials can utilize sound to boost sensing. This work has potential applicability in environments with significant ambient noise, such as transportation hubs, for the design of security screening or pollution monitoring devices. Where the peptide materials can also potentially be used with traditional piezoelectric materials^29,30^ to enhance energy harvesting device performance.

## Data Availability

The data that supports the findings of this study are available within the article.

## Supporting Information Available

Details of the materials and methods section and additional spectroscopy and imaging data (PDF)
