# Supplementary material for: Piezoelectric Peptide Nanotube Substrate Sensors Activated through Sound Wave Energy
Source: ACS Mater Lett. 2024 Apr 8;6(5):1863–9. doi: 10.1021/acsmaterialslett.3c01613 (PMC11077579; doi:10.1021/acsmaterialslett.3c01613)
Supplement: Supplementary file 1 — tz3c01613_si_001.pdf [file tz3c01613_si_001.pdf]

Piezoelectric peptide nanotube substrate sensors activated through sound wave energy.

Sawsan Almohammed,[1,2] Allan Finlay,[1] Dominik Duleba,[3] Shane Cosgrave,[1] Robert Johnson,[3] Brian J. Rodriguez,[1,2,]* James H. Rice,[1,]*

[1]School of Physics, University College Dublin, Belfield, Dublin 4, D04 V1W8, Ireland
[2]Conway Institute of Biomolecular and Biomedical Research, University College Dublin, Belfield, Dublin 4, D04 V1W8, Ireland
[3] School of Chemistry, University College Dublin, Belfield, Dublin 4, D04 V1W8, Ireland

Materials and characterisation methods

Preparation of surface-patterned electrode substrates

Si substrates (100 orientation, Si Mat) were cleaned of surface contaminants by dipping in acetone for 2 minutes, washing with ethanol and isopropanol, rinsing in deionised water, and drying using nitrogen.[6] The mask, comprising pieces of a Si substrate cut using a diamond scribe, was placed in contact with a Si substrate during exposure to UV/ozone (ProCleaner, BioForce) for a specified duration, allowing an oxide layer to grow on the unmasked Si surface . A mask with an opening size of 0.2 cm was then added to the substrate. In lieu of cleaning, some samples were etched using hydrofluoric acid (48%) for 60 seconds to remove the native oxide, washed in deionised water, and blown dry using nitrogen. The etched samples were subsequently stored in ethanol and blown dry using nitrogen prior to UV/ozone exposure. In order to fabricate a wettability gradient, the substrates were selectively exposed to UV/ozone for 10 through the mask openings. Following this a second mast was added that covered the silicon oxide region and gold electrodes where deposited using vapour deposition.

Preparation of peptide nanotube stock solution

Stock solutions of peptide nanotube were prepared by dissolving the L-diphenylalanine peptide (Bachem, Bubendorf, Switzerland) in 1,1,1,3,3,3-hexafluoro-2-propanol at an initial concentration of 100 mg/ml that was further diluted in deionized water to final concentrations of 4, 2, and 0.5 mg/ml. New solutions were prepared for each alignment experiment.

Alignment of peptide nanotubes

20 µl of peptide nanotube solution was pipetted onto the centre of the central masked region of the SiO2 substrate. In some cases, prior to deposition onto a room temperature substrate, the 2 mg/ml peptide nanotube solution was heated at 100°C for 5 minutes, at which point the solution turned from opaque to clear, a phenomenon previously reported by Huang et al. and attributed to the reversible disassembly of the PNTs.

Coating peptide nanotubes with Asilver nanoparticles

After heating at 100°C for 5 minutes, 90 µl of 2 mg/ml peptide nanotube solution was added to a 10 µl aqueous solution of 0.02 mg/ml 20 nm diameter silver nanoparticles (Ag NPs) (795933, Sigma-Aldrich), which was also heated at 100°C for 5 minutes prior to mixing. The combined

solution was stirred for 3 minutes and then 20 µl was deposited on the room temperature Si substrate that was exposed to UV/ozone for 20 minutes using a mask with 0.5 cm openings.

Scanning electron microscopy

Scanning electron microscopy (SEM) (JSM-7600F, JEOL, operated at 5 kV) was performed to characterise the morphology of the PNTs. Before imaging, a thin (~ 8 nm) layer of gold was sputtered on the substrates (Hummer IV, Anatech USA). The diameters of the peptide nanotube were determined from SEM images. Each value reported is the average diameter of 20 peptide nanotubes.

Optical microscopy

Optical micrographs (10x objective) were used to quantify the alignment of the peptide nanotubes. The radial summation of the fast Fourier transform (FFT) of each image was determined using ImageJ and the radial profile plug-in (National Institutes of Health). The full width at half max (FWHM) of each of the two peaks in the resulting plot of sum versus angle was determined by fitting to a Gaussian function using OriginPro 8.5.1 (OriginLab). For all samples studied, the mean FWHM and standard deviation were calculated typically from 6 images (i.e., 12 peaks), 3 from each mask opening, to account for slight variations in the placement of the drop on the centre of the central masked region.

Raman spectroscopy

SERS measurements were performed using a bespoke Raman system comprising an inverted optical microscope (IX71, Olympus). In brief, a monochromatic laser (HeNe, ThorLabs) with beam splitter and long pass filter (RazorEdge, Semrock), a spectrograph (SP-2300i, Princeton Instruments), and a CCD camera (IXON, Andor). A 50 X objective was used to focus the laser (532 nm wavelength, 5 mW incident power) and collect the Raman signal with an exposure time of 1 s.

Piezoelectric measurements of peptide nanotubes

Piezoresponse force microcopy (PFM) was implemented using an Asylum Research MFP 3D AFM and n-type Si probes with Pt coating (HQ:CSC37/Pt, Mikromasch) with a nominal stiffness of 0.3 N/m and nominal resonance frequency of 20 kHz. A 10 kHz, 30 V AC voltage, which had been amplified using a voltage amplifier (F10A, FLC Electronics AB), was applied to the AFM probe and a lock-in amplifier (HF2LI, Zurich Instruments) was used to measure the in-plane piezoresponse amplitude and phase signals.

Measuring piezoelectric output during substrate bending

A Keithley 6514 electrometer with a LabVIEW interface was used to measure open-circuit voltage and short-circuit current. Average current and voltage values are determined from a total of 30 measurements, 10 measurements (peaks) from three samples.

Characterization of peptide nanotube-silver nanoparticle composite

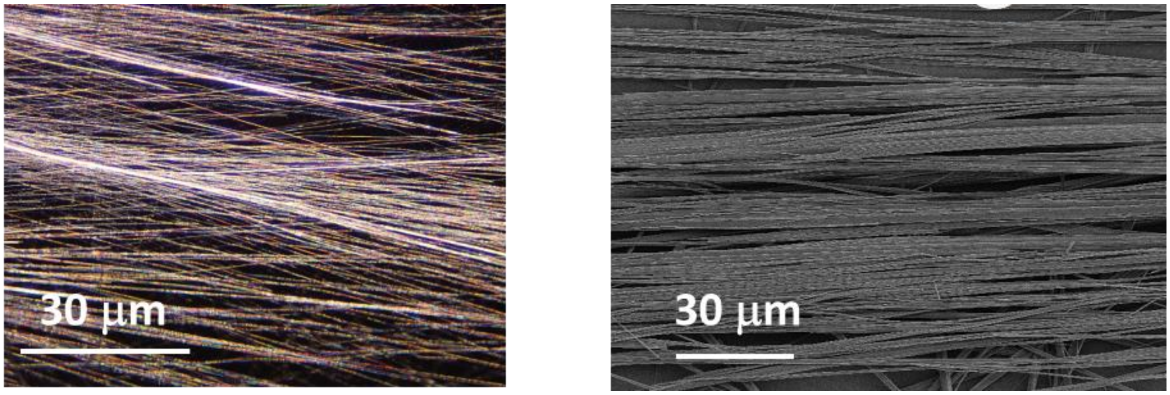

Fig. S1. Imaging of aligned peptide nanotubes. (a) Optical image of aligned peptide nanotubes. For comparison (b) shows a scanning electron microscopy (SEM) image of the aligned peptide nanotubes recorded with similar image resolution.

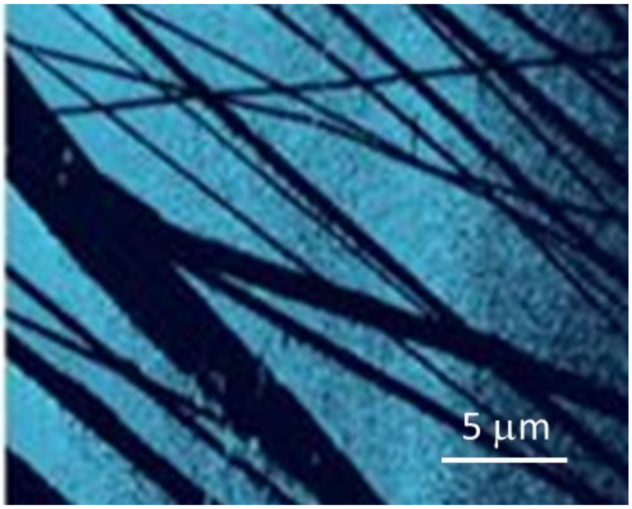

Fig S2. Lateral Piezoresponse force microscopy (LPFM) phase image of aligned peptide nanotubes

Lateral piezoresponse force microscopy (LPFM) phase images of the peptide nanotubes were recorded. The PFM phase images provide information on the polarization direction of the PNTs (D. Denning, J. Guyonnet, and B. J. Rodriguez, Int. Mater. Rev. 61, 46 (2016)). The piezoelectric polarization is related to the molecular ordering in the peptide nanotubes. It is known that the piezoelectric polarization observed in peptide nanotubes is correlated with the specific molecular

ordering and arrangement of the peptides within the nanotube structure (Li, Tong, Xian-Mao Lu, Ming-Rong Zhang, Kuan Hu, and Zhou Li. "Peptide-based nanomaterials: Self-assembly, properties and applications." *Bioactive Materials* 11 (2022): 268-282). The LPFM phase response can be considered in-phase or out-of-phase concerning the driving voltage and depends on the orientation of the PNT and thus the sign of the shear piezoelectric coefficient. Fig S2 shows the nanotubes as the dark contrast as being in-phase. The nanotubes can be seen to deform in-phase and out-of-phase (dark and bright contrast in Fig S2). The high degree of uniformity of polarisation indicates strong alignment and a high degree of crystallinity in the peptide tube.

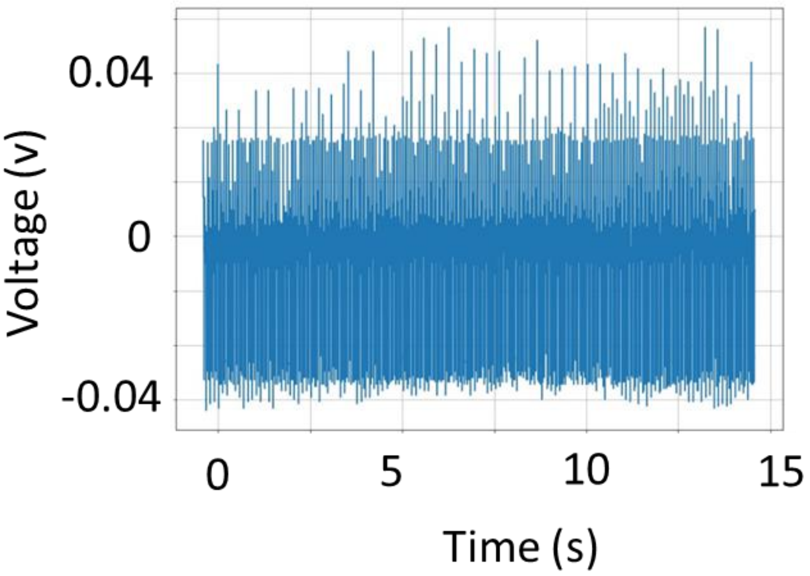

Fig. S3. Plot of voltage vs time. This was recorded for the silicon oxide substrate with gold electrodes in the absence of any peptide nanotubes to show the background voltage output from the system. This will be much lower than the voltage output from the active PENG design (shown in Fig.1 of the main paper) which has peptide nanotubes present. These piezoelectric nanotubes have been central to achieving the voltage output of > 1V.
